# Molecular identification and genotyping of hepatitis E virus from Southern Punjab, Pakistan

**DOI:** 10.1038/s41598-023-50514-5

**Published:** 2024-01-02

**Authors:** Muhammad Muddassir Ali, Mehek Gul, Muhammad Imran, Muhammad Ijaz, Shahan Azeem, Arif Ullah, Hafiz Muhammad Farooq Yaqub

**Affiliations:** 1 Institute of Biochemistry and Biotechnology, Faculty of Biosciences, University of Veterinary and Animal Sciences, Lahore, Pakistan; 2 Department of Veterinary Medicine, Faculty of Veterinary Science, University of Veterinary and Animal Sciences, Lahore, Pakistan; 3 Institute of Microbiology, Faculty of Veterinary Science, University of Veterinary and Animal Sciences, Lahore, Pakistan

**Keywords:** Microbiology, Molecular biology, Zoology

## Abstract

Hepatitis E is a global health concern. Hepatitis E virus (HEV) infection is endemic in Pakistan. HEV has four genotypes: HEV-1 through HEV-4. The genotypes HEV-1 and HEV-2 are associated with infection in humans, especially in countries with poor sanitation. The genotypes HEV*-*3 and HEV-4 are zoonotic and human infection takes place by consuming undercooked meat or being in contact with animals. The present study was designed to ascertain the presence of HEV in the Southern Punjab region of Pakistan. First, blood samples (n = 50) were collected from patients suspected of infection with the hepatitis E virus from the Multan District. The serum was separated and the samples were initially screened using an HEV IgM-ELISA. Second, the ELISA-positive samples were subjected to PCR and were genetically characterized. For PCR, the RNA extraction and complementary DNA synthesis were done using commercial kits. The HEV ORF2 (Open Reading Frame-2, capsid protein) was amplified using nested PCR targeting a 348 bp segment. The PCR amplicons were sequenced and an evolutionary tree was constructed using MEGA X software. A protein model was built employing the SWISS Model after protein translation using ExPASy online tool. The positivity rate of anti-HEV antibodies in serum samples was found as 56% (28/50). All Pakistani HEV showed homology with genotype 1 and shared common evolutionary origin and ancestry with HEV isolates of genotype 1 of London (MH504163), France (MN401238), and Japan (LC314158). Sequence analysis of motif regions assessment and protein structure revealed that the sequences had a similarity with the reference sequence. These data suggest that genotype 1 of HEV is circulating in Pakistan. This finding could be used for the diagnosis and control of HEV in the specific geographic region focusing on its prevalent genotype.

## Introduction

Hepatitis E is a disease of worldwide medical importance. The disease is caused by the hepatitis E virus (HEV) which induces acute viral hepatitis. Viral hepatitis ranges from asymptomatic infection of the liver to jaundice, diarrhea, nausea, and fever leading to high morbidity and mortality in immune-compromised people. In the immune-compromised people, the disease manifests as a chronic condition with extra-hepatic manifestations such as renal disorder, neurological problems, and hematological diseases^1^. HEV is a single-stranded positive-sense RNA virus belonging to the genus *Hepevirus* of the family *Hepeviridae*^2^. Acute hepatitis caused by HEV is self-limiting and resolves in two to six weeks^2^. The number of HEV-related infections is estimated at 20 million per year, with 3 million symptomatic cases and around 70,000 deaths annually. Although these figures are extremely large, they still represent a gross underestimation of the true global burden of disease^3^. The virus is transmitted via the fecal–oral route through contaminated water or food^4^. The incubation period for HEV is ~ 2–9 weeks^5^.

HEV has eight genotypes of which only four are involved in causing diseases in humans: HEV-1 through HEV-4^6^. The HEV-1 and HEV-2 are found commonly in developing countries including Africa, Asia, and Mexico. The HEV-3 and HEV4 are common in developed countries^6^. Genotypes 1 and 2 are associated with infection in humans and outbreaks commonly occur in countries with poor sanitation^7^. Genotypes 3 and 4 are transmitted by the consumption of undercooked meat or possibly being in contact with animals. The HEV strains originating from rabbits, pigs, camels, and rats have zoonotic potential^8^. The HEV is found worldwide but commonly reported in East and South-East Asian countries^9^. The HEV infection is endemic in developing countries. Moreover, HEV is thought to be the most common cause of acute sporadic hepatitis in Pakistan^10,11^. In many underdeveloped countries, the incidence of HEV in pregnant women is very high with mortality rates ranging from 20–30%^12^. In Pakistan alone, > 15% of pregnant women were diagnosed with the hepatitis E virus, all of them with no history of jaundice^13^. Owing to the impact on the young, productive population and the pregnant women, HEV infections of humans warrant investigations. The presence of HEV has been studied in other Asian countries including Bangladesh, India, Iran, China, Nepal, and Japan^14^. Scant data on HEV genetic characterization from Pakistan are available. However, serological evidence of HEV has been found in Pakistan. To our knowledge, this is the first report of detection and genetic characterization of human HEV from the Southern Punjab region of Pakistan. The genetic characterization of HEV could have implications for the epidemiology, diagnostics, and vaccines. The present study was aimed at the identification and genetic characterization of the human HEV from the Multan District of the Southern Punjab region of Pakistan.

## Materials and methods

### Study design and sample collection

The present study was designed for the identification and genotyping of HEV from the human samples collected from Multan City of the Southern Punjab region of Pakistan. The Southern Punjab region was selected because of the low literacy rate and poor sanitary conditions in the area: factors likely to favor HEV transmission. The data were collected from people who voluntarily agreed to participate by completing an informed consent form. For this purpose, a questionnaire was developed and completed before collecting blood samples from patients. The questionnaire consisted of the following information: name, age, gender, living status, drinking, and eating habits, and the history of other hepatitis: HAV (Hepatitis A Virus), HBV (Hepatitis B Virus), and HCV (Hepatitis C Virus) or jaundice (Supplementary Materials). Sample collection (n = 50) and all other methods were carried out in accordance with relevant guidelines and regulation and the study was approved by the Institutional Review Committee for Biomedical Research, University of Veterinary and Animal Sciences, Lahore, Pakistan (with Approval letter No. 104/IRC/BMR, 01/10/2021.

From each of the fifty HEV-suspected patients, 5–10 ml blood was collected in gel clot activator tubes (Biota, Istanbul, Turkey) and the serum was separated within 6 h of sample collection. The collected serum was stored at − 20 °C till further processing.

### ELISA

The serum samples were initially screened using HEV-IgM ELISA Kit (Bioss, Woburn, MA, USA) following the manufacturer's instructions. Briefly, 10 µl of serum sample along with 100 µl of sample dilution buffer was added into the wells of an ELISA plate. The optical density was measured at the wavelength of 450 nm using AMP Micro Plate Reader (Agilent, Santa Clare, CA, USA). The positive and negative results were declared based on absorbance as A > 0.8 and A < 0.1. The ELISA-positive samples were processed for viral RNA extraction.

### RNA extraction

The viral RNA was extracted from 200 µl of serum using the TRIzol method following the manufacturer’s instructions in the Institute of Biochemistry and Biotechnology, University of Veterinary and Animal Sciences, Lahore, Pakistan. Two hundred microliters serum sample was admixed with TRIzol. After mixing and short incubation, the same volume of chloroform was added and centrifuged at 13,000 rpm for 15 min. Upper layer was separated and mixed with Isopropanol in equal volume. After centrifugation, the supernatant was discarded and the pellet was collected. To the pellet, 300 µl chilled ethanol was added and incubated on ice. Pellet was dried and 100 µl sterile distilled water was added in pellet for further use. The quantity of RNA was determined by a Nanodrop 2000 Spectrophotometer (Thermo Fisher Scientific, Waltham, MA, USA). Complementary DNA (cDNA) was synthesized using Revert Aid First Strand DNA Synthesis Kit (Thermo Fisher Scientific, Waltham, MA, USA) following the manufacturer’s instructions.

### PCR amplification

The PCR amplification was performed with two sets of primers targeting the ORF2 gene of HEV using a nested PCR approach. The following primer pairs were designed using Primer3 software (V.0.4.0): Outer primers; RfF1 (5′-GCCGAGTATGACCAGTCCA-3′), RfR1 (3′-ACAACTCCCGAGTTTTACCC-5′); Inner primer; RfF2 (5′-AATGTTGCGACCGGCGCGC-3′), RfR2 (3′-TAAGGCGCTGAAGCTCAGC-5′) for Pakistani HEV isolates. The PCR was performed in two successive rounds. The first round of PCR was performed using the outer set of primers in a 25 µl reaction and the following reaction mixture: 0.5 µl RfF1 forward primer, 0.5 µl RfR1 reverse primer, 1 µl template cDNA, 2 µl dNTPs, 0.25 µl *Taq* polymerase, 2.5 µl PCR buffer, 0.5 µl MgCl_2_, and 17.75 µl deionized water. The cycling conditions for the first round of PCR were: Initial denaturation at 95 °C for 5 min, 40 cycles of denaturation at 94 °C for 30 s, annealing at 53 °C for 30 s, and extension at 72 °C for 30 s, followed by the final extension at 72 °C for 5 min.

The second round of PCR was performed using the inner set of primers also in 25 µl and using the following reaction mixture: 0.5 µl RfF2 forward primer, 0.5 µl RfR2 reverse primer 1 µl amplicon of the first round of PCR as a template, 2 µl dNTPs, 0.25 µl *Taq* polymerase, 2.5 µl PCR buffer, 0.5 µl MgCl_2_, and 17.75 µl deionized water. The cycling conditions for the second round of PCR were: an initial denaturation at 95 °C for 5 min, 35 cycles of denaturation at 94 °C for 30 s, annealing at 53 °C for 30 s, and extension at 72 °C for 30 s, followed by the final extension at 72 °C for 5 min.

### DNA sequencing analysis

The PCR products were run on 2% agarose prepared in 1X TAE buffer, stained with ethidium bromide and an amplicon of 348 bp was visualized with a 1 kbp ladder using a UV Gel Documentation System (Bio-Rad, Hercules, CA, USA). After the purification of samples by 80% ethanol, the samples were sequenced using a DNA Genetic Analyzer 3500xL (Applied BioSystems, Thermo Fisher Scientific, Waltham, MA, USA) via a commercial vendor. The sequenced DNA samples were analyzed using various bioinformatics tools. The sequences were aligned using the Basic Local Alignment Search Tool (blast.ncbi.nlm.nih.gov/) and compared with reference sequences available in the National Center for Biotechnology Information (NCBI) data bank. Chromas software (2.6) was used to visualize and analyze the sequencing results. For correct coding region, different open reading frames were analyzed for protein analysis. For this purpose, DNA sequences of different sample sequences were translated into putative amino acid sequences using ExPASy tool. The alignment of all protein sequences was done by Clustal Omega Multiple Sequence Alignment Tool. A phylogenetic tree was constructed using Molecular Evolutionary Genetic Analysis (MEGA-X) using Neighbour Joining Method. For motif detection, Multiple Expectation Maximization (MEME) Suite software was used for motifs identification in a group of related protein sequences. Protein structure was built by SWISS Model (Supplementary Fig. 3a, b, and c). The highly matched sequence in FASTA format was used to build model in phylogenetic tree each branch was labeled with virus accession number along with the geographic area from where the virus was isolated. DNA sequences of different samples were translated into protein (supplementary Fig. 2a, b, and c) using the ExPASy tool available free online (https://web.expasy.org/translate/). Different open reading frames were analyzed for the correct coding region.

## Results

### The positivity rate of anti-HEV antibodies in serum samples

Out of 50 serum samples collected from 2019 through 2021 from HEV-suspected patients, 56% (28/50) samples tested positive for anti-HEV antibodies.

### The positivity rate of HEV RNA in ELISA-positive samples

ELISA-positive samples were processed for viral RNA extraction and out of 28 ELISA-positive serum samples; HEV RNA was detected in 15 (53.57%) samples.

**Figure 1:**
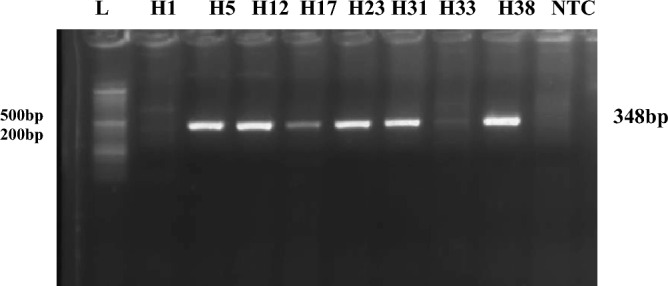
HEV samples by agarose gel electrophoresis, where L represents Ladder (1kbp), NTC is negative control and the rest are HEV samples.

A nested PCR targeting ORF2 gene of HEV demonstrated that the annealing temperature of 53 °C demonstrated optimum amplification as at this temperature, no non-specific amplification was observed. The product size was approximately 348 bp. The gel picture is shown in Fig. 1 and supplementary Fig. 1.

### Phylogenetic analysis

All positive PCR products (n = 15) were sequenced and the cleaned sequences were deposited to the NCBI GenBank database with the following accession numbers: MZ474606, MZ474607, MZ474608, MZ474609, and MZ474610. All these sequences were used for phylogenetic analysis. The sequenced Pakistani HEVs aligned with reference sequences of HEV genotypes available in the GenBank database. Among study population of Pakistan, HEV genotype 1 demonstrated to be the predominant one as all the sequences obtained in the present study were similar to HEV genotype 1.

**Figure 2:**
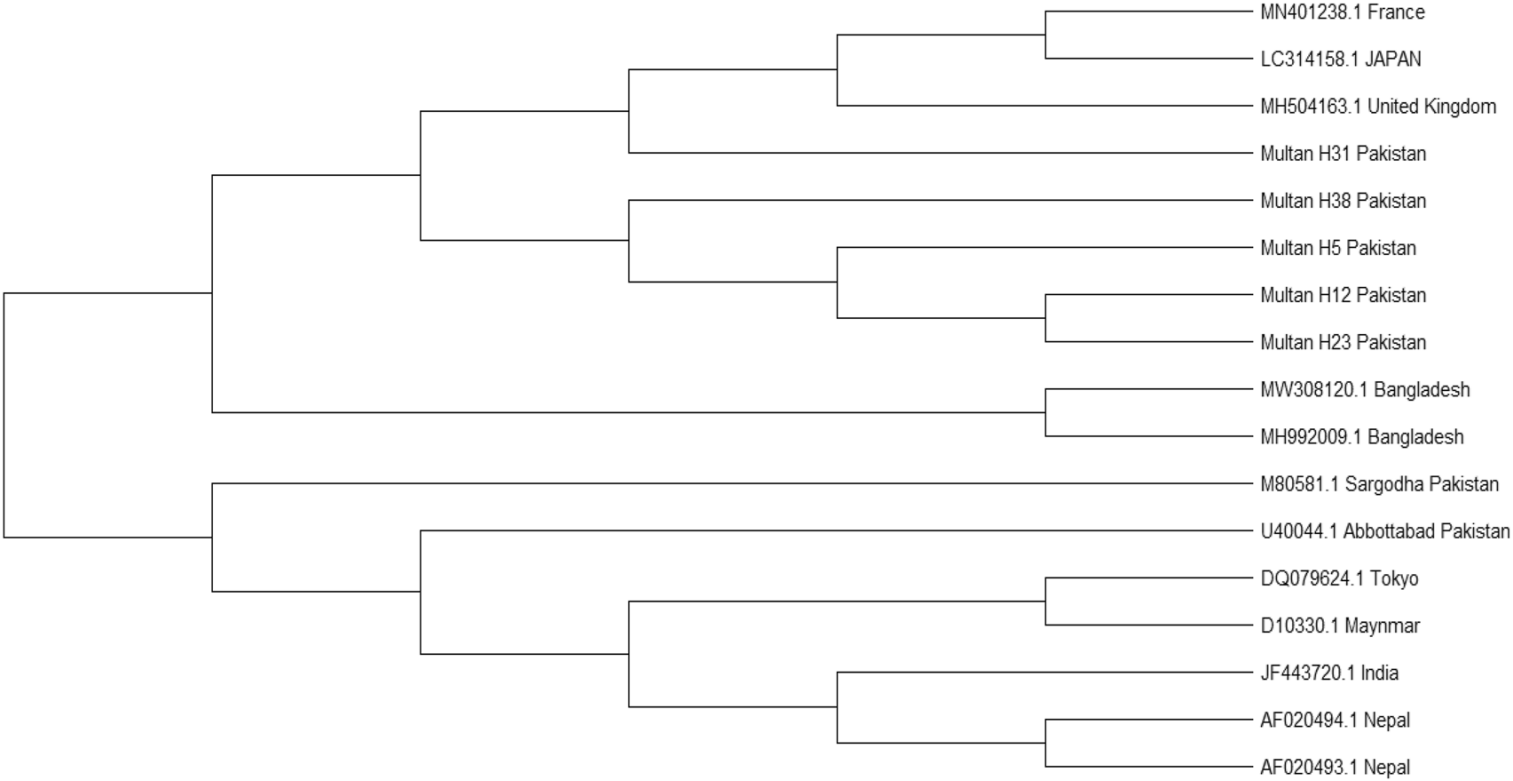
Phylogenetic tree of HEV genotype 1 (ORF2) constructed by MEGA X using Neighbor-Joining method.

The phylogenetic tree was constructed using reference sequences of genotype 1. One sample sequence (MZ474606) lied close to genotype 1 of London (MH504163.1), while others (MZ474607, MZ474608, MZ474609, and MZ474610) belonged to the same cluster and demonstrated close homology to each other. The phylogenetic tree is shown in Fig. 2.

### Motifs location

**Figure 3:**
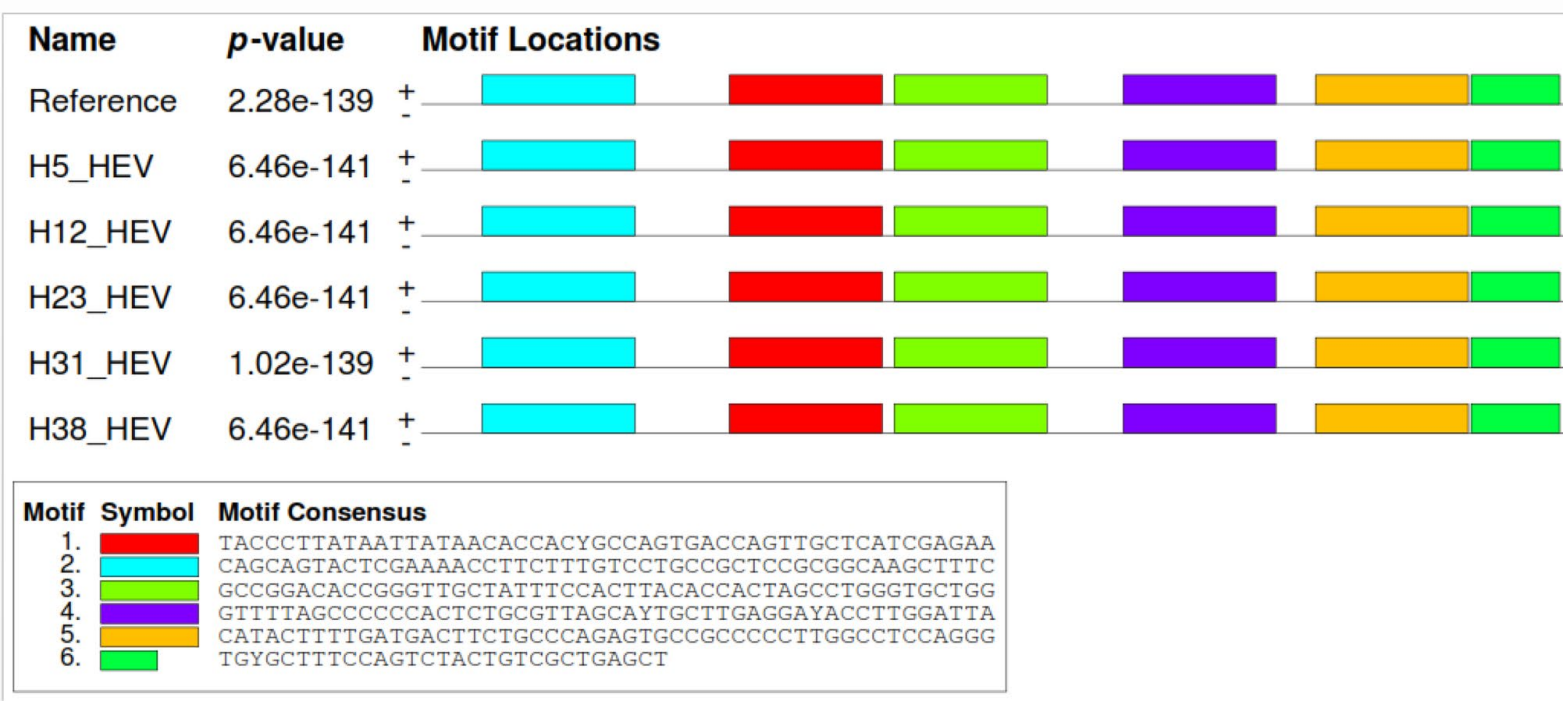
Nucleotide motif locations that show sequence similarity between different ORF2 HEV sequences.

**Figure 4:**
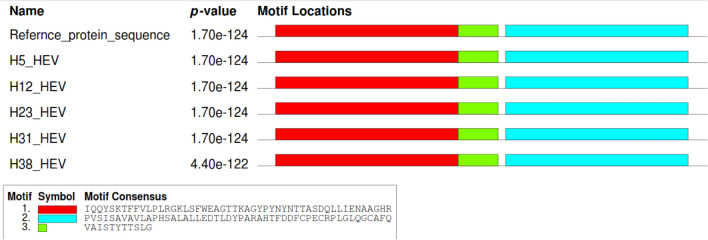
Protein motif locations which show amino acid sequence sharing between different ORF2 capsid protein sequences of HEV.

The nucleotide sequences of DNA and amino acid sequences of protein were processed through MEME Suite software version 5.5.0, available free online (https://meme-suite.org/meme/) for motif locations. The motif locations showed that HEV sequences shared similar motifs in all the samples and those motifs matched with reference sequences (MH504163.1). The location of motifs from nucleotide and protein sequences is shown in Figs. 3 and 4, respectively.

### Multiple sequence alignment

**Figure 5:**
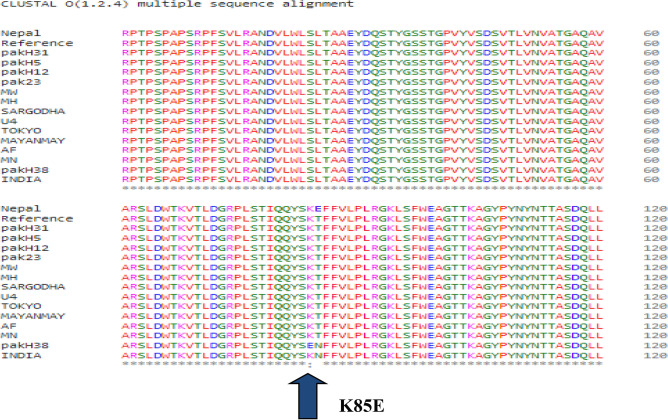
Multiple Sequence Alignment showing mutations between HEV sequences of capsid protein.

All the protein sequences were aligned using Clustal Omega which showed amino acid changes in the H38-HEV sample compared with all other sequences of other countries and reference (MH504163.1) protein sequences. The mutations within the sequence are shown in Fig. 5. The K (Lysine) amino acid of the reference protein sequence was substituted with the E (Glutamic Acid) amino acid of the H38-HEV sample at 85th position (K85E). The sequence from Nepal also showed variation at a single amino acid change from the reference sequence, T (Threonine) to E (Glutamic acid) at 86th position (T86E).

## Discussion

Hepatitis E virus infection is a major medical issue worldwide that seriously affects the health and productivity of human beings. The present study was conducted to evaluate the presence of and genetically characterize HEV in the Multan City, Southern Punjab, Pakistan. To our knowledge, the present study is the first one to report the presence of HEV and genetically characterize HEV from Southern Punjab, Pakistan. In the present study, the positivity rate of anti-HEV antibodies was 56% in human serum samples collected from 2019 through 2021. Of the ELISA-positive serum samples, HEV RNA was detected in 15 (53.57%) samples. Genetic analysis of the HEV detected in the present study demonstrated that genotype 1 is predominantly circulating in Sothern Punjab region of Pakistan. The present study has implications for HEV diagnostics and vaccine production.

Several methods for amplification of the ORF2 gene have been reported, as the ORF2 gene is commonly used to characterize and genotype HEV strains. In Hong Kong, the Hepatitis E Virus-C1 genotype was detected in 0.27% blood samples collected from 2,860 patients suffering from abnormal liver functions. Three patients demonstrated an acute phase of hepatitis^15^.

The presence of anti-HEV antibodies and HEV RNA in humans in China was found to be 22.68 and 0.41%, in India 27.15 and 6.59%, in Iran 8.98 and 0.24%, respectively^16^. According to one study in France, the seroprevalence of hepatitis E is 52% and the incidence of the disease is 3%^17^.

In the present study, nested PCR was carried out which is likely to increase the sensitivity and specificity. There is a limitation due to serological detection method in immune-compromised people. The percentage of HEV-positive samples is high in the HEV RNA detection approach. Therefore, PCR assays are used for the determination of HEV infection during the acute phase and in immune-compromised patients, due to the absence or delayed production of antibodies. In Switzerland, the genotype-3 subtype was detected in patients with acute hepatitis by PCR method^15^.

The detection of all four genotypes of the hepatitis E virus is a difficult and challenging due to the mutations in virus strains^18^. Therefore, sequence analysis is compulsory for the identification of novel genotypes. The chain termination sequencing method was performed for ORF2. This method involves the encoding of capsid protein and binding of 5′ of the HEV genome^19^. In the current study, the phylogenetic analysis based analysis of 348 bp of ORF2 region, revealed that all the Pakistani HEV samples showed homology with HEV genotype 1 sequences from the NCBI databank. The sample sequences showed more than 95% sequence identity with each other but more than 90% sequence similarity with other HEV genotype 1 of different countries. The HEV genotype 1 is prevalent in Asian countries where virus infection is associated with monsoon rains and poor hygienic conditions^14^. In addition, genotype 1 and genotype 2 are associated with disease in humans. HEV is considered hyperendemic in developing countries^20^. Mutations in the glycosylation sites of the ORF proteins of HEV capsid has been observed. Mutations in this region may play a role in the severity of the disease in terms of antibody escape, vaccine break, increased transmissibility, and increased host cell permeability. This site plays an important role in virus-host interactions and thus important for the spread of viral infection across host species^21–23^.

The prevalence of hepatitis E in the Pakistani population is ~ 20–22% in adults and ~ 14–26% population is exposed to HEV^14^ Genotype 1 seems responsible for acute hepatitis infection in adults and pregnant women with mortality rates of 1% and more than 30%, respectively^16,24^. Like many other developing countries, the prevalence of HEV in pregnant women in Pakistan is very high^25^. However, the reasons for the higher mortality rates, especially in the third trimester, are not determined. It is suggested that pregnant women must be screened regularly as it is necessary for the health of mother and fetus^26^. In Pakistan, HEV genotype 1 with isolates Sar-55 (87-Pakistan-A), Abb-2b (88-Pakistan-2B), and 87-Pakistan-B is dominant^26^. The spread of HEV on unhygienic conditions such as poor waste management systems, leakage of water pipes, and the use of untreated water on agricultural land. Floods due to heavy rain in Pakistan could also have led to an increased incidence of HEV^11^. The incomplete removal of HEV from water sources can lead to outbreaks^27^. In the case of water treatment methods, boiling water used for drinking purposes is an effective treatment for reducing the level of Hepatitis E Virus in areas where water is highly contaminated with HEV^26^. It was first suggested that chlorination can reduce the level of Hepatitis E Virus, but later it was reported that Ultraviolet disinfection is one of the most important water disinfection method that damages HEV genome by creating photo dimers^26^. Pakistan is a developing country with poor sanitary conditions and a lack of proper waste management systems. The wastewater is a major source of the spread of the virus directly draining into rivers and other water reservoirs during rainy seasons^4^. Many developments have been reported in the fight against infectious diseases. Now World Health Assembly is trying to work on Global Health Sector Strategy to reduce or eliminate viral hepatitis. The main objective of this strategy is to reduce the number of hepatitis cases from 6 to 10 million to less than a million as well as to reduce the death from > 1 million to 0.5 million^28^. The present study’s identification of region-specific HEV- genotype could help in the development of targeted intervention strategies.

One limitation of the present study was the small sample size from a specific geographic region of Pakistan. Another limitation was the absence of age-wise positivity rate comparison, which could not be performed due to difficulty in the availability of samples from various age groups. The role of environmental factors in the spread of HEV was not included in the present study’s evaluations. Despite these limitations, the present study’s results provide baseline data on the presence of HEV in the Southern Punjab region of Pakistan. The present study confirmed and phylogenetically characterized HEV from Southern Pakistan. Future studies exploring the presence of HEV in other regions of Pakistan and their molecular characterization could further increase our understanding of the HEVs circulating in Pakistan.

## Conclusion

HEV is common in the Southern Punjab region of Pakistan. Genotype 1 of HEV was found to be the most common genotype in the studied region. Moreover, genotype identification in a geographical area may have key implications in developing region-specific diagnostics and vaccines.

### Supplementary Information

Supplementary Information.

## Data Availability

The sequences were submitted to NCBI database resource with following Accession Numbers: MZ474606, MZ474607, MZ474608, MZ474609, and MZ474610. Additional data will be made available on justified demand.
