# Supplementary material for: Molecular identification and genotyping of hepatitis E virus from Southern Punjab, Pakistan
Source: Sci Rep. 2024 Jan 2;14:223. doi: 10.1038/s41598-023-50514-5 (PMC10762251; doi:10.1038/s41598-023-50514-5)
Supplement: Supplementary file 1 — Supplementary Information. [file 41598_2023_50514_MOESM1_ESM.pdf]

Supplementary Materials

Molecular Identification and Genotyping of Hepatitis E Virus from Southern Punjab, Pakistan

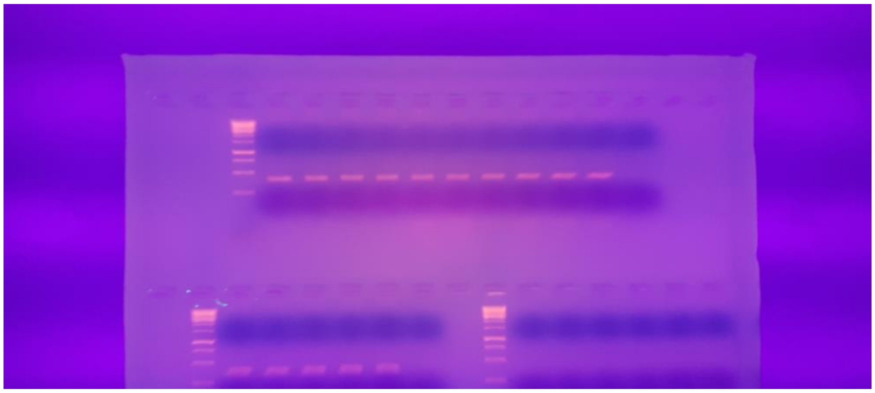

Supplementary Figure 1. Original Gel picture, an unprocessed version.

Protein Analysis

The protein-translated sequences are shown in (suppl. Fig. 2a, 2b, and 2c).

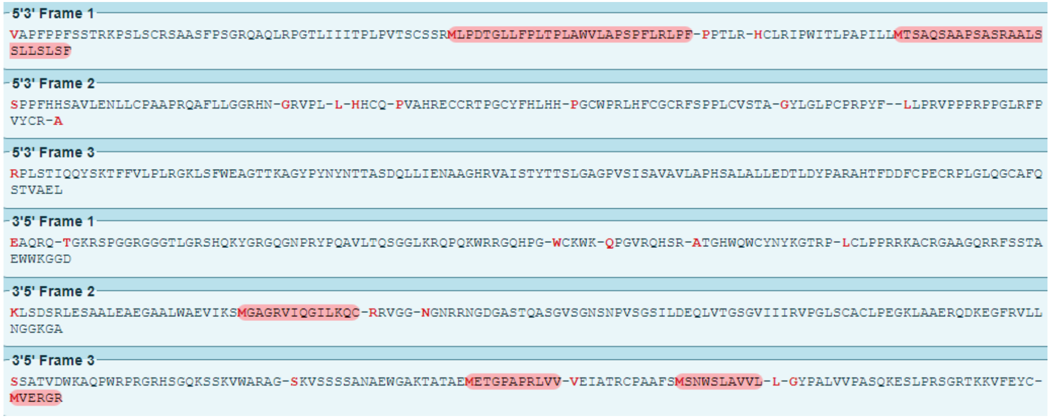

5'3' Frame 1

VAPFPPFSSTRKPSLSCRSAASFPSGRQAQLRPGTLIIITPLPVTSCSSRMLPDTGLLFPLTPLAWVLAPSPFLRLPF-PPTLR-HCLRIPWITLPAPILLMTSAQSAAPSASRAALS
SLLSLSF

5'3' Frame 2

SPPFHHSAVLENLLCPAAPRQAFLLGGRHN-GRVPL-L-HHCQ-PVAHRECCRTPGCYFHLHH-PGCWPRLHFCGCRFSPPLCVSTA-GYLGLPCPRPYF--LLPRVPPPRPPGLRFP
VYCR-A

5'3' Frame 3

RPLSTIQQYSKTFFVLPLRGKLSFWEAGTTKAGYPYNYNTTASDQLLIENAAGHRVAISTYTTSLGAGPVSISAVAVLAPHSALALLEDTLDYPARAHTFDDFCPECRPLGLQGCAFQ
STVAEL

3'5' Frame 1

EAQRQ-TGKRSPGGRGGGTLGRSHQKYGRGQGNPRYPQAVLTQSGGLKRQPQKWRRGQHPG-WCKWK-QPGVRQHSR-ATGHWQWCYNYKGTRP-LCLPPRRKACRGAAGQRRFSSTA
EWWKGGD

3'5' Frame 2

KLSDSRLESAALEAEGAALWAEVIKSMGAGRVIQGILKQC-RRVGG-NGNRRNGDGASTQASGVSGNSNPVSGSILDEQLVTGSGVIIIRVPGLSCACLPEGKLAAERQDKEGFRVLL
NGGKGA

3'5' Frame 3

SSATVDWKAQPWRPRGRHSGQKSSKVWARAG-SKVSSSSANAEWGAKTATAEMETGPAPRLVV-VEIATRCPAAFSMSNWSLAVVL-L-GYPALVVPASQKESLPRSGRTKKVFEYC-
MVERGR

Supplementary Figure. 2a. Protein translation by ExPASy (a) Reference Protein

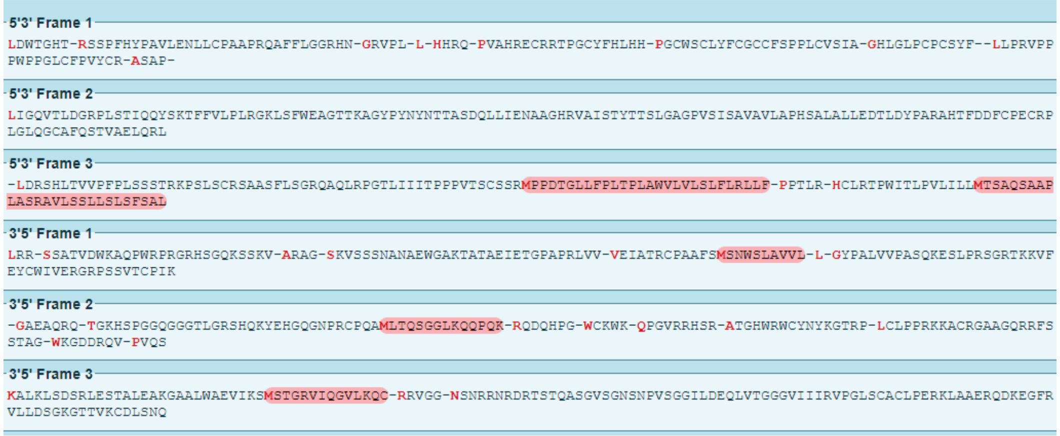

Fig. 2b. Protein translation by ExPASy (b) H12-HEV sample

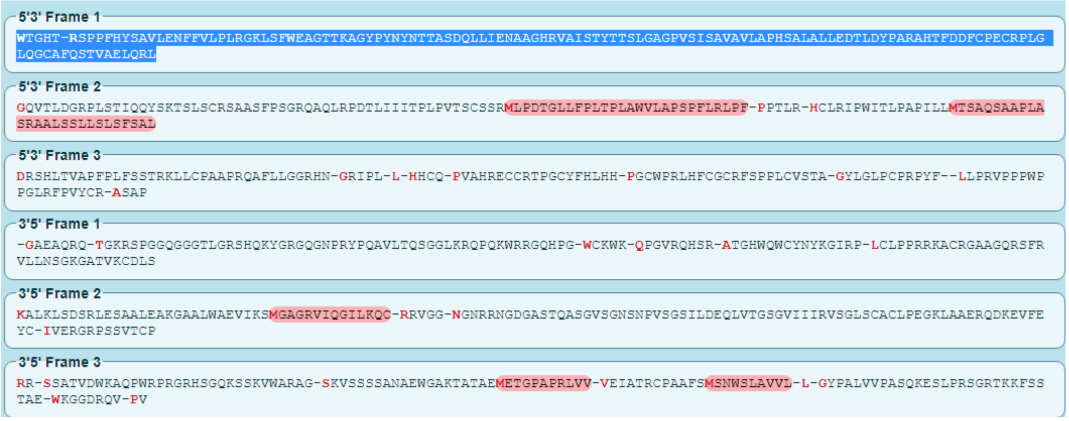

Supplementary Figure. 2c. Protein translation by ExPASy (c) H38-HEV sample

Modeling of protein structure

The open reading frames were processed through p-BLAST for homology with HEV sequences. The highly matched sequence was used for the SWISS Model to build protein structure. The protein structure is shown in suppl. Fig. 3a, 3b, and 3c.

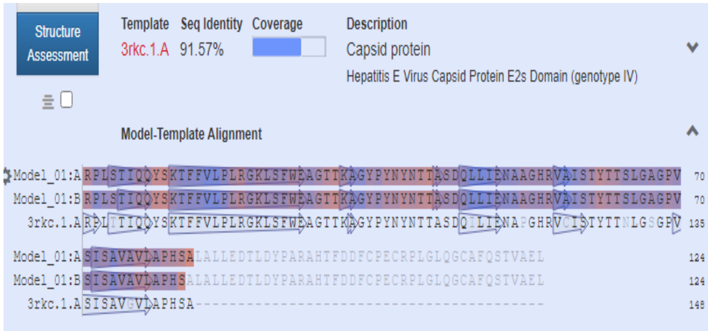

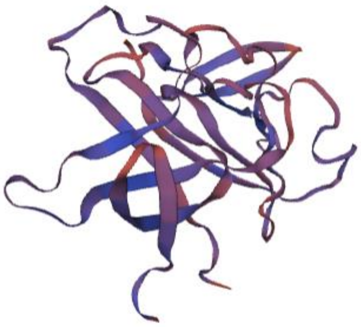

Supplementary Figure. 3a. Hepatitis E virus Capsid protein (a) Reference protein sequence

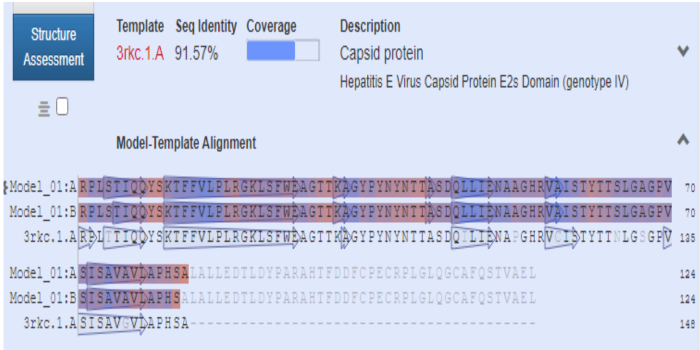

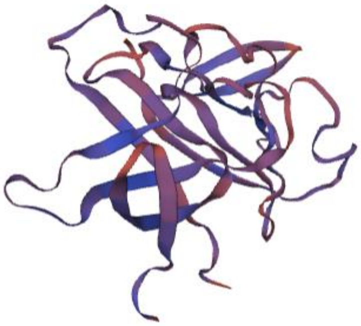

Supplementary Fig.3b. Hepatitis E virus Capsid protein (b) H5, H12, H23, and H31 - HEV samples

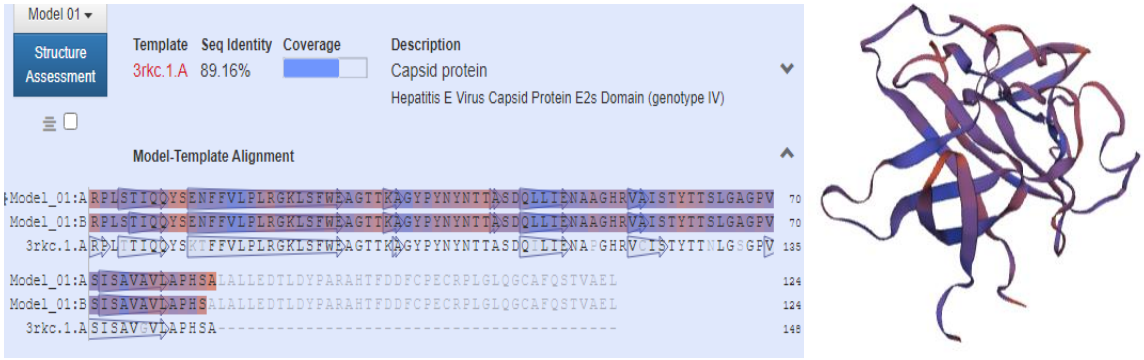

Supplementary Fig. 3c. Hepatitis E virus Capsid protein (c) H38-HEV sample

QUESTIONARE

QUESTIONNAIRE

Institute of Biochemistry and Biotechnology (IBBT)
University of Veterinary & Animal Sciences, Lahore

Research Title:

Molecular identification and genotyping of *Hepatitis E virus* from Southern Punjab Pakistan

Sample ID: ________________

Name:____**Age:** ________________________

- ☐ Male
- ☐ Female

Symptoms:

- ☐ Present
- ☐ Absent

History of other hepatitis:

- ☐ Anti-HAV IgG
- ☐ HBV HBsAg
- ☐ Anti-HCV
- ☐ Anti-HDV

Consent for Participation in Study

I have been explained about the research project by the researcher. I have understood given information and I am donating my blood to use solely for the purpose of genetics research and dissemination if necessary.

Subject

Name:_________________________Signature/Thumb Print: -----------------------------
